# Parental obligations, care and HIV treatment: How care for others motivates self-care in Zimbabwe

**DOI:** 10.1177/1359105318788692

**Published:** 2018-07-20

**Authors:** Morten Skovdal, Rufurwokuda Maswera, Noah Kadzura, Constance Nyamukapa, Rebecca Rhead, Alison Wringe, Simon Gregson

**Affiliations:** 1 University of Copenhagen, Denmark; 2 Biomedical Research and Training Institute, Zimbabwe; 3 Imperial College London, UK; 4 London School of Hygiene & Tropical Medicine, UK

**Keywords:** antiretroviral therapy, care, family, HIV, obligation, Zimbabwe

## Abstract

This article examines how parental obligations of care intersect with HIV treatment-seeking behaviours and retention. It draws on qualitative data from eastern Zimbabwe, produced from 65 interviews. Drawing on theories of practice and care ethics, our analysis revealed that norms of parental obligation and care acted as key motivators for ongoing engagement with HIV services and treatment. Parents’ attentiveness to the future needs of their children (*caring about*), and sense of obligation (*taking care of*) and improved ability to care (*caregiving*) following treatment initiation, emerged as central to understanding their drive for self-care and engagement with HIV services.

## Introduction

Advances over the past decade, fuelled by global commitments, resources and innovation in the fight against HIV and AIDS, have led to extraordinary progress in the delivery of HIV services to low-resource and high HIV prevalence regions of the world. In sub-Saharan Africa, the number of people on antiretroviral therapy (ART) has doubled in the past 6 years, reaching nearly 10.3 million people in 2016 (UNAIDS, 2016a), contributing to significant declines in HIV-related deaths. Pooled analyses of population-based datasets from sub-Saharan Africa have indicated mortality declines following the expansion of ART at rates between 58 per cent and 84 per cent, depending on the population (Reniers et al., 2014).

Progress, however, should not overshadow the many persistent challenges that continue to threaten global targets to end AIDS by 2030. Although 10.3 million people have been enrolled onto ART in eastern and southern Africa, this figure still only makes up 54 per cent of people in the region who need treatment (UNAIDS, 2016a). There is a continued need to improve the supply of HIV services to different populations. However, challenges pertaining to HIV treatment adherence and retention (Fox and Rosen, 2010) suggest that antiretroviral treatment programmes depend not only on the supply of HIV services but also on the demand and motivation of people living with HIV to self-care and engage with HIV services (Russell et al., 2016). While there is a burgeoning body of research unpacking the bottlenecks to HIV care engagement (Hardon et al., 2007; Skovdal et al., 2011a; Wringe et al., 2017), with much focus on the supply side, relatively little has been done to understand the demand for, and pathways to, HIV care (cf. Martin et al., 2013; Russell and Seeley, 2010; Skovdal et al., 2011b). This article sets out to make a conceptual and empirical contribution to our understanding of how social relationships support engagement with HIV services specifically, and self-care more generally.

### Social relationships, familial responsibilities and living with HIV

While there is plenty of research looking at how norms of family obligation play an instrumental role in availing care and support for people living with, and affected by, HIV (Ankrah, 1993; Iwelunmor et al., 2008; Knight et al., 2016; Masquillier et al., 2014; Seeley et al., 1993; Skovdal et al., 2009), little has been done to unpack how norms of obligation, as they fall upon people living with HIV, shape their motivation to engage with treatment practices. A particularly noteworthy study is the ethnographic work done by Ware et al. (2009) in Nigeria, Uganda and Tanzania. In an effort to understand why adherence rates in sub-Saharan Africa are generally better than levels of adherence observed in many North American settings (Mills et al., 2006), Ware and colleagues investigate the role of social relationships in antiretroviral treatment. They found social relationships to play a facilitating role, both by availing different forms of resources, such as food, cash and encouragement, and by conferring a sense of responsibility. Ware et al. (2009) observed that individuals on antiretroviral treatment, because of the support they received from their social networks, often felt a sense of obligation to prevent a deterioration of their health – and thereby lighten the burden of their helpers – motivating adherence to antiretroviral treatment. A feedback loop, with helpers recognizing the adherence efforts of individuals on antiretroviral treatment, was noted to further strengthen social relationships and ensure support in the future (Ware et al., 2009). This notion of social responsibility, or obligation for others, has also emerged as central to the lived experiences of people living with HIV in China. A phenomenological study of 21 individuals living with HIV in northern China found that conceptions of the possibility of a premature death created a sense of guilt among some participants, due to a fear they may not be able to fulfil their family obligations, either as a carer for their elderly parents, or by being a parent for their children (Zhou, 2010). Family obligation also emerged as a theme in an interview study with 16 individuals living with HIV and five family members in the Shaanxi province of western China (Zhang et al., 2013). Rather than seeing family obligation as a resource, the study highlights the flipside of social relationships, where people living with HIV, because of their perceived obligation and care for family members, work long hours and moderate their daily lives to protect and prioritize the needs of their family (Zhang et al., 2013). Ware et al. (2009) also observed such ‘social coercion’ (cf. Binagwaho and Ratnayake, 2009), with people living with HIV making sacrifices, such as going without food, for family members.

It is clear from this emerging literature that social relationships, norms of family obligation and care, play an instrumental role in shaping the lived realities of people living with HIV. In this article, we build on this important work by examining how parental obligations of care intersect with HIV treatment-seeking behaviours and retenion.

## Conceptual framework

To disentangle the relationship between parental obligations of care and engagement with HIV services, we draw on two analytic constructs. The first one is *practice theory*, which provides a productive lens for examining the relationship between practices, such as care and HIV treatment practices. Schatzki (2010) and Shove et al. (2012) speak of *bundles of practices* to describe an assemblage of co-dependent practices and highlight the importance of understanding how practices, through their connections, may co-evolve, collaborate or compete for resources. If our interest is to understand what it takes for people living with HIV to join, maintain or defect from engagement with HIV treatment, we need to understand what and how other life practices, such as care, intersect with this particular practice. This insight can be used to form or break, strengthen or weaken links between them (Blue et al., 2016), with the aim of enhancing engagement with HIV treatment (Skovdal et al., 2017).

We also draw on the concept of *care ethics* to make visible the interdependent care practices and interactions that are mobilized in the ‘local’, whether at home, or in the community, and structure relationships that promote mutuality and wellbeing (Lawson, 2007). The concept of care ethics challenges traditional understandings of care, as someone either being a carer or cared for, and sheds light on different care interactions, including reciprocities of care. Lawson (2007) and Tronto (1993) argue that care ethics are central to our interactions and encounters with others and involves values of empathy, responsiveness, attentiveness and responsibility that are exchanged interdependently. Tronto (1993) distinguishes between four forms of care, each of which allows us to unpack specific care dynamics, interactions and norms of parental obligation:

1. ‘caring about’, associated with attentiveness to the needs of others;
2. ‘taking care of’, associated with responsibility, which is culturally constructed;
3. ‘caregiving’/‘care for’, associated with competence to provide ‘good care’, including the availability of adequate resources (materiality);
4. ‘care-receiving’, associated with the responsiveness of the care-receiver to the care (Tronto, 1993: 131–136).

Although gender norms and constructions of care are contingent on the socio-historical and cultural context of a given society, care is often devalued as feminized and private work (Tronto, 1993). We will apply a gender lens to our data, in order to gauge whether gendered understandings of care are at stake. In bringing these analytical constructs together, we offer a framework for exploring how interactions, in their different manifestations of care, intersect with the practice of engaging with HIV services and treatment.

## Methods

Data were generated from a larger qualitative interview study (the ‘bottlenecks’ study) that sought to locate differentiated experiences of engagement with HIV care services in the context of people’s lived realities (see Wringe et al., 2017). The Bottleneck’s study was granted ethical approval by the London School of Hygiene and Tropical Medicine (Ref: 10389) and the Medical Research Council of Zimbabwe (MRCZ/A/1990). Informed and written consent was obtained from all participants with the agreement that anonymity was upheld. We have therefore used pseudonyms throughout.

### Study location and participants

This study was conducted in the Manicaland province of Eastern Zimbabwe. Manicaland reached very high HIV prevalence rates in the late 1990s, with a peak of 24 per cent between 1998 and 2000 (Gregson et al., 2006). The Manicaland province has since witnessed a steady decline in HIV prevalence, in part due to reductions in sexual risk behaviour, reaching a level of 15 per cent between 2009 and 2011 (Gregson et al., 2017). There are however significant spatial differences in the province, with small towns and their surrounding areas still experiencing HIV prevalence rates in excess of 25 per cent, despite their closer proximity to HIV services (Schaefer et al., 2017). Since the introduction of the public sector ART programme in 2004, national HIV treatment coverage has steadily increased from 4 per cent in 2004 to 62 per cent in 2015 (UNAIDS, 2016b).

Participants for the study were recruited from five sites, representing small towns, roadside trading centres and subsistence farming villages. All sites are characterized by high levels of poverty and HIV. We interviewed people living with HIV (*n* = 59) and family members of people who had died from AIDS-related illnesses (*n* = 6). People living with HIV were recruited through local health clinics and HIV support groups, while family members of deceased were identified from previous verbal autopsy interviews. Participants were purposefully recruited to represent a broad distribution of sex, age and diagnosis and care histories, including those diagnosed but not initiated on ART (*n* = 16); initiated but not retained (*n* = 8); on ART for at least 6 months (*n* = 35) and deceased (*n* = 6).

### Qualitative data collection and analysis

Experienced Shona-speaking qualitative researchers (including R.M., N.K., C.N.) conducted the interviews using semi-structured topic guides. Topic guides were tailored to the type of participant being interviewed. Topics guides for people living with HIV sought to elicit differentiated experiences of (dis)engagement with HIV testing, care and treatment services and place these in the context of their lived realities. The interviews included a health journey tool, which involved the interviewers noting down visually the respondents’ experiences of using different types of health services, including the approximate date when things happened. Interviews with family members of the deceased sought to understand the circumstances that led to their death.

Interviews were conducted in the preferred language of the participant, and at a private venue preferred by the participants. This was often in a room in the local health clinic, or at a local school during out of hours. The interviews were conducted between October 2015 and April 2016 and lasted between 45 and 90 minutes each. With consent from the participants, we digitally recorded the interviews. All interviews were anonymized and transcribed into English, with all files kept in password protected folders. The transcripts were subsequently imported into qualitative software package NVivo 11 for coding, thematic organizing and exploration. This was done by M.S. who followed the steps of Attride-Stirling’s (2001) thematic network analysis to develop a broad analytical coding framework. This involved an inductive process of first coding the transcripts, applying interpretive titles (child nodes) to text segments. The codes were subsequently clustered together into more interpretive basic themes (parent nodes), which in turn were grouped into organizing interpretive categories (grandparent nodes). As not all of the themes are of relevance to this article, we only draw on data coded, and thematically organized, under the heading ‘Motivations to engage with HIV services’. This subset of data was subsequently subject to a more analytical and interpretive interrogation (by all authors), aided by our theoretical framework and driven by our interest to disentangle the data that spoke to the relationship between norms of family obligation, parental care and motivation to engage with HIV services.

## Findings

On the basis of our thematic analysis we organize and present our findings to demonstrate how parental obligations of care intersect with self-care.

### Care for others motivates self-care

Practices of self-care and engagement with HIV services were by many of our, particularly female, participants motivated by different forms of care for their children. A number of women spoke about how ART had enabled them to live productive lives, making it possible for them to perform their role as a mother, and *care for* their children again:

ARVs are good. They are good because they allow me to live. I tested positive in 2002, which means I have lived with the virus for nearly 15 years, and I am still alive. I have four children who are negative. ARVs are good because they allow you to live a normal life. You can be there for your family as long as you are taking ARVs. It is good to have a family and they will be helping you. It helps you to have children. (Jane, age 40, living with HIV)

This is also exemplified by Melissa, who is a widow and mother of three. When asked what her participation in HIV care has done for her, she responded:

I am well and able to look after my three orphaned children. If I want to go to the fields I am able to plough without feeling any pain. I no longer wait for people to assist me. (Melissa, age 32, widow, living with HIV, mother of three)

Melissa is able to care for her children and no longer relies on charity from others. Care was often expressed as a form of responsibility. Twenty-nine-year-old Celestine, for example, spoke about how ART had enabled her to *take care of* her children and live up to her obligation as a parent. She spoke about her treatment in relation to how it had helped her regain trust in life and her productive-self and finds motivation in nurturing a future where she will get grandchildren:

Just because someone is positive does not mean you are useless in life. It does not mean you cannot take care of your child. Right now my child is six but he can still grow up and marry and I can be able to see my daughter in law and grandchild even though am positive. (Celestine, age 29, widow, mother of one)

A couple of fathers expressed similar sentiments. Peter, for example, who has also witnessed the difference ART makes to his health, notes that HIV care has enabled him to *take care of* his family. Being healthy, and able to live up to his obligation as a father and a husband, was for Peter a strong motivating factor of self-care, so much so, that it, according to him, helps him resist discouraging advice from others, and encourages him to recruit others into HIV care:

Haaa, people do talk about me, but I feel the effects of HIV care in my life. My life is progressing and I am able to take care of my family. I will not listen to people who try to dissuade me from visiting the clinic. In fact, I encourage others. (Peter, age 46, married, father of four)

For some women, care was expressed through imaginaries of what life would be like for their children if they were no longer alive and around to take care of them. This attentiveness to the future needs of their children (*caring about*), fuelled by perceptions that children would suffer following the passing of both parents, was a key motivator for many of our female participants to engage with HIV care services. This is exemplified by 40-year-old Alice, who shortly after her husband’s death to AIDS-related illness, got urgently enrolled onto ART. When asked what motivated her to get tested and initiate treatment, she responded:

People on antiretroviral therapy are surviving. If I stop [taking the medication] I will die and leave my children orphaned. Their father is already dead, and for me to also die will leave the children to suffer. So I accepted to work with the present situation. (Alice, age 40, widowed, living with HIV)

Knowing the life-saving potential of ART, having witnessed the impact of HIV and AIDS on her husband, and her *caring about* the future of her children, made Alice self-care by accepting her diagnosis and persistently taking her medication. For many of our female participants, being alive for their children meant safeguarding their education and giving them hope for the future. Joyce, for example, when reflecting back on the news of her sero-conversion, re-called an immediate concern about the education of her three boys:

When my results came, and I was told I was positive that got me worried. I started to think of what I will do, my life, my children that got me worried because my children are still young so if I fell sick how were they going to go to school, who will provide money for school fees, food and all that. So right now I am better. (Joyce, age 35, divorcee, living with HIV)

Alice and Joyce’s imaginary that orphaned children are left to suffer and will struggle to stay in school, is, in the absence of public welfare, not wholly unfounded. Mercy, a grandmother who has assumed the caregiving role of her orphaned grandchildren, states clearly the difficulties she is facing in taking her grandchildren to school. She notes the importance of children having at least one living parent, cementing a form of ‘social coercion’:

Mmmm I can say my problem because everyone has his or her problem. I can say when John died the problem I have is that of taking care of the children. I am old and can no longer go to do part time jobs to sustain this family. This is when I think that if the parents were here or at least if one parent was alive it could have been better because they would look after them and be able to go to school. Sending them to school is actually a problem because I do not have the money. This is what is troubling me. (Mercy, age 70, mother of a son who passed away from AIDS-related illness)

Some of our female participants, who spoke about the importance of staying alive to care for their children, took scenarios, like those described by Mercy, into account and extended their *caring about* to the wider community. They saw it as their obligation to raise the children, and not to trouble or burden others with this responsibility. One participant spoke about how she did not want her orphaned children to become troublemakers, making life difficult for people in the community. These examples demonstrate how imaginaries of what life would be like if they were not around and able to care for their children, intersect with their motivation to engage with HIV services, improve health and prolong life.

However, not all study participants experienced a smooth transition into ART. A number of participants experienced severe side-effects as a result of their antiretroviral treatment, preventing them from living normal, productive and caring lives. One participant, Jacquie, made a conscious decision to stop her treatment, explaining that her treatment and associated side-effects prevented her from being a parent and *care for* her children:

2014. I was finally given ARVs. My face, hands and legs started swelling. My health was deteriorating and I was not in a position to support my family. I am both the mother and father so I decided to stop. (Jacquie, age 54, widow, mother of five)

As the quote alludes to, Jacquie is widowed and the sole carer of her children. In order to be in a position to care for her children, Jacquie made the difficult decision to stop her HIV treatment, exemplifying how care for others, in some circumstances, can also prevent self-care.

Care was not unidirectional, but characterized by reciprocities. We observed numerous instances where parents on ART received care from their children (see also the quote by Jane above). This *care-receiving* took many different shapes and forms, but was often projected in the interviews as gentle reminders from their children, and spouses, about taking their medicines:

*What support do you get?* My children and husband support me because they witnessed the problems that I encountered before taking the tablets. They saw how it affected me, so at the moment they remind me to take my medication. It is also because of the change to my health that they are witnessing. (Patricia, age 33, mother of five children, living with HIV)

Our qualitative data suggest that the practice, engagement with HIV services (self-care), was for many of our participants intrinsically interwoven with the practice of caring for others. ART enabled parents to be parents. In most cases, the two practices not only existed in harmony but actively supported each other, facilitating HIV care. Only in a few rare circumstances, such as the one described by Jacquie, did the practice of engaging with HIV services and self-care conflict with the practice of being a caring parent, resulting in treatment drop out.

## Discussion

Our findings point to factors that motivate engagement with HIV services specifically and self-care more generally. Improved access to ART made it possible for our participants to live, care for others, and perform their social responsibilities, such as being a parent or a spouse, which in turn sparked engagement with HIV services. Witnessing the transformative impact of ART helped our participants overcome fears of a premature death and not being able to care for loved ones, fears that have also been noted in China (Zhou, 2010) and the United States (Ingram and Hutchinson, 2000). Specifically, our analysis suggests that being a mother, and in a few instances, being a father, and their attentiveness to the future needs of their children (*caring about*), and sense of obligation (*taking care of*) and improved ability (*caregiving*) to care, emerged as key motivators to engagement with HIV services and treatment. Although others have highlighted the role of social relationships in facilitating adherence to ART, as far as we are aware, this is the first study to examine how parental obligations of care support engagement with HIV services. Relatedly, a recent study by Wamoyi et al. (2017) drawing on interviews with PLHIV in Malawi, South African and Tanzania, noted how the anticipation of one day becoming a parent, strengthened ongoing engagement with HIV services.

**Figure 1.**
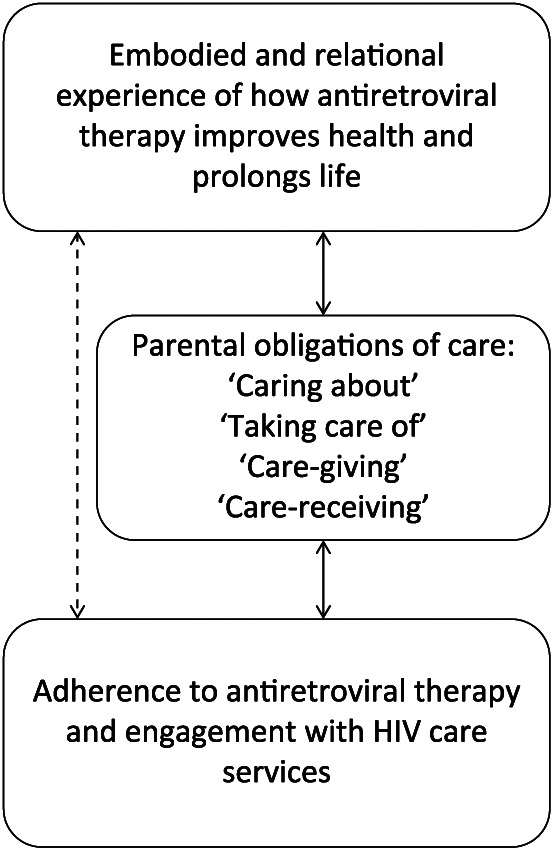
Mediating role of care ethics and familial responsibilities to engagement with HIV care services.

Our study builds on the seminal work of Ware et al. (2009) in four ways. First, although we did not frame the study around the concept of social capital, our study extends their hypothesis that social capital, understood as care and support interactions within a network, is associated with better access and adherence to ART. Second, by introducing the concept of ‘care ethics’, we have been able to disentangle new and multiple forms of care, responsibility and norms of parental obligation that motivate engagement with HIV services and self-care. Care ethics also encourages us to recognize the mutuality of care interactions, namely that care for others leads to self-care, providing a care spin to the ideas that social relations and norms of family obligation in Africa ‘push’ people to engage with HIV services and treatment (Binagwaho and Ratnayake, 2009). Third, the vast majority of text segments presenting themes relevant for this article came from female respondents. This indicates that more women than men are able to utilize care ethics and parental obligations of care as a resource and motivator to engage with HIV services and treatment. This may potentially offer one explanation for women’s superior engagement with HIV services and treatment compared to men (Abioye et al., 2015; Boullé et al., 2015). It should be said, however, that the gender differences noted in this study, although benefitting women, may reflect repressive gender relations. Studies of motherhood in the context of HIV infection highlight that mothers living with HIV often face stigma and have no time to be HIV positive, working tirelessly to place their family first (Ingram and Hutchinson, 2000; Kanniappan et al., 2008). Fourth, our findings also revealed the important role of positive embodied and relational experiences of ART in unfolding care interactions and self-care. For many of our participants, having witnessed both the devastating impact of HIV and the health-enabling potential of ART was a strong motivator to engage with HIV services, not least if it meant they were able to fulfil their parental obligations of care. While others have also noted the importance of witnessing the transformative effects of ART for engagement with HIV services (Horter et al., 2017; Renju et al., 2017), we observed care for others, manifested in parental obligations of care, to be particularly strong mediators in cementing this association (see Figure 1).

Our findings are constrained by some methodological limitations, which deserve mentioning. First, the generalizability of our findings is limited and may not apply to other settings, as our geographic and cultural environment may well vary from others. Second, our study relies on self-reported data and it is difficult for us to ascertain what happens in practice. As a consequence, our study was susceptible to social desirability bias, such as participants presenting themselves as good and responsible parents. A conceptual limitation of the study is our inability to differentiate between voluntary and coercive care. We fully recognize the, at times, coercive nature of care and familial responsibilities, particularly among women, something we have not been able to consider in this article.

## Conclusion

It was not uncommon for our participants, and in particular our female participants, to talk about illness or treatment experiences through care interactions and family obligations. Through their accounts, we have examined the multiple and complex ways in which parental obligations of care intersect with adherence to antiretroviral therapy and engagement with HIV services and care. We observed that people living with HIV, who have responsibilities of care, appear to find meaning in their care interactions, which has the potential to motivate and support engagement with HIV services. Our study provides a new perspective on how social relationships can support engagement with HIV services and treatment. We also observed the enabling capacity of positive embodied and relational experiences of ART. For many of our participants, the transformative potential of ART made it possible for them to conceive a caring role, and effectively a reason to engage with HIV services and treatment.

Our findings suggest that the practice of caring for others can facilitate the practice of engaging with HIV services. This observation can be used to explain possible variances and differences in engagement with HIV services and treatment, for instance between men and women. Our findings speak to the important role of teleological projects, such as raising children, in shaping experiences and engagement with HIV services and treatment. Future research needs to examine how having an apparent purpose, directive principle or goal, whether that involves care ethics and familial responsibilities or not, shape treatment experiences and successes.
